# Multiband terahertz metamaterial perfect absorber for microorganisms detection

**DOI:** 10.1038/s41598-023-46787-5

**Published:** 2023-11-11

**Authors:** Ruchi Bhati, Anil K Malik

**Affiliations:** Photonics and Metamaterials Laboratory, Department of Physics, Chaudhary Charan Singh University, Meerut, Uttar Pradesh 250004 India

**Keywords:** Optics and photonics, Physics

## Abstract

We report a multi-resonant terahertz (THz) metamaterial perfect absorber (MPA)-based biosensor in the working frequency range of $0 - 3.8 THz$ for sensing of microorganisms (such as fungi, yeast) and wheat pesticides. Nearly $100\%$ absorption is realized at $f_1= 1.7THz, f_2= 2.8THz, f_3=3.2THz,$ and $f_4=3.5THz$. We designed our THz MPA sensor making resonators’ gap area compatible with the microorganisms’ size. To obtain optimum performance of the MPA, a mapping of amplitudes and shifts in the absorption resonance peaks with different structural parameters of the resonators is carried out. A very high-frequency shift is obtained for microorganisms such as Penicillium chrysogenum (fungi), yeast, and pesticides (Imidacloprid, N, N-Diethyldithiocarbamate sodium salt trihydrate, Daminozide, N, N-Diethyldithiocarbamate sodium salt hydrate, and Dicofol). An equivalent circuit model using Advance Design System (ADS) software is developed. The calculated results through the model show similar trends as obtained in the simulations using CST. Investigations of the effect of incidence angle of THz wave on the absorption spectra of the MPA are also carried out. It is found that incidence angle does not impact the stability of the lower resonance absorption peak (1.79*THz*). Due to the wide working frequency range, the proposed sensor is extremely suitable for the detection of all range of pesticides because their specific absorption fingerprint lies in the frequency range of 0–3.8*THz*. We believe that our sensor could be a potential detection tool for detecting pesticide residues in agriculture and food products. The THz MPA-based biosensor is capable of detecting a very small change in the effective dielectric constant of the MPA environment. Therefore, it can also offer huge opportunities in label-free biosensing for future biomedical applications.

## Introduction

Sensors are a vital part of modern technology due to their applications in imaging, medicine, food quality control, agriculture, defense etc. Food safety and security are two foremost public health concerns that require fast and non-destructive inspection techniques^1–4^. Due to fingerprints of pesticide and antibiotics in terahertz (THz) spectral range^5, 6^, THz spectroscopy facilitates non-contact, label-free, non-destructive inspection for food safety and control^7–12^. Moreover, early detection of microorganisms such as bacteria, viruses, and fungi is also very important for food security^13, 14^. However, due to the typical size of $\lambda /100$, microorganisms are transparent at THz frequency leading to low scattering cross-section and are difficult to detect by the THz spectroscopy^15^. To overcome this constraint, metamaterial-based THz sensors have been proposed for the detection of microorganisms like bacteria, yeast, and molds^16^, where metamaterial resonators are designed in such a way that the gap area of resonators becomes compatible with the size of microorganisms. Here, the resonance frequency of metamaterials is extremely sensitive to any changes in the dielectric constant of the gap area of the resonators^17, 18^. Extensive research is being carried out to develop metamaterial-based resonant devices^19–21^. For example, Hossain et al. proposed photonic crystal fiber-based sensor for petrochemical sensing applications^22^ and detection of bane chemicals^23^. Among these devices, metamaterial perfect absorbers (MPAs) can offer huge opportunities due to their potential applications^24, 25^.

It is possible to minimize reflectivity and transmittivity close to zero for MPAs by impedance matching. To further expand the application prospects of MPAs, the number of absorption frequency peaks and workable frequency bands need to be broadened. Several reports show that flexibility to work in a wide range of frequency bands can be obtained by optimizing the dimensions of MPAs^26–32^. Khan et al.^33, 34^ proposed a technique to design a THz absorber for biosensing applications specially for the detection of breast cancer, malaria in blood and glucose in water by altering the multimodal resonance on ultrathin silicon ring resonator in frequency range of 5–8THz. Varshney et al.^35^ numerically analyzed a graphite/dielectric cavity resonator-based absorber for wideband absorption. Zamzam et al.^36^ designed an MPA with a metal-dielectric-metal multi-layer column with different heights of the dielectric layer, where they achieved two absorption peaks. Wang et al.^37^ demonstrate a triple-band terahertz metamaterial absorber at 0.337, 0.496, and 0.718THz where they used three concentric square ring resonators. A metamaterial absorber consisting of three closed circular ring resonators on a polyethylene terephthalate substrate is investigated by Abdulkarim et al.^38^, where a Q-factor of 50.72 was reported. Meng et al.^39^ demonstrated a multi-band terahertz absorber made of periodic square metallic ribbon with a T-shaped gap. Several resonator structures like square spiral shape^40^, SSRR( square split ring resonator)^41^, Jerusalem Cross^42^ , CSRR (Circular split ring resonator)^43^, eight-resistive arm cell^44^, and Ring C-shape quasi-MMA^45^ have been investigated. Most of the reported THz meta-sensors suffer from limitations such as larger unit cell size, one or two absorption peaks, low absorption coefficients, and polarization sensitivity. A small range of working frequency of a sensor limits it to the detection of some specific pesticides and microorganisms while, a wide working frequency range of a sensor can help in detecting a wide range of pesticides and microorganisms. The maximum working frequency range reported earlier lies between $0.4 -2.4THz$ which can not cover all ranges of pesticides and microorganisms($0-4THz$). Moreover, multiple resonances enhance the flexibility of the sensor.

In this paper, we report a THz metamaterial perfect absorber-based biosensor creating resonators’ gap area compatible with the microorganisms’ size. We obtain almost $100 \%$ absorption at $f_1= 1.7THz, f_2= 2.8THz, f_3=3.2THz,$ and $f_4=3.5THz$ in THz frequency range between $0-3.8 THz$ due to strong inductive–capacitive coupling of resonators. This wide working frequency range of MPA is highly required for fast and non-destructive detection of microorganisms like molds, yeast cells, fungi, and pesticides because their fingerprints lie in the same frequency range. Our investigations are the first to detect microorganisms and wheat pesticides using THz MPA. We carry out detailed simulations to study the sensing performance of the proposed sensor. We obtain the frequency shift 103*GHz* (high sensitivity) for microorganisms and 95*GHz* for pesticides. A mapping of shifts in the resonance peaks of MPA with structural parameters is also carried out. Interference theory is used to explain the absorption mechanism. An equivalent circuit model using Advance Design System (ADS) software is developed. Multi-resonant MPA-based THz biosensors are remarkably desirable for label-free biosensing of a family of microorganisms (bacteria, yeast, molds, etc.). Authors believe that the proposed sensor is a potential detection tool for detecting pesticide residues in agriculture and food products.

## Design and analysis of MPA based sensor

**Figure 1:**
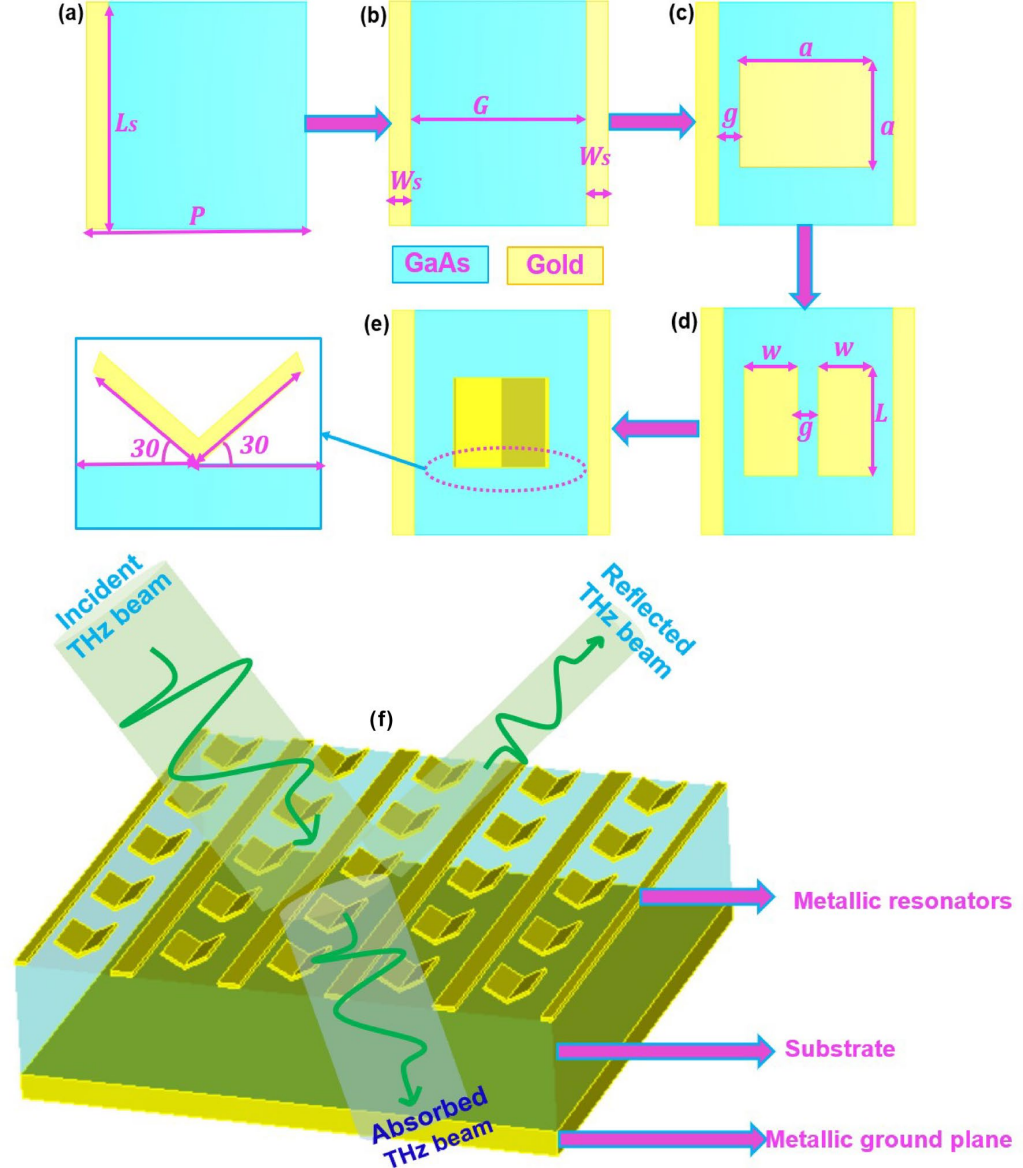
Schematic of the unit cell with (**a**) only one metal stripe resonator, (**b**) two metal stripes resonators, (**c**) two stripes + one square resonator (SR), (**d**) two stripes + two SRs, (**e**) two stripes + 1 open book resonator, which is designed by putting two SRs obliquely, and (**f**) a schematic of MPA - based sensor with incident, reflected, and absorbed THz beam.

We investigate several metastructures consisting of (1) only one stripe resonator, (2) two stripes resonator, (3) two stripes and one square resonator (SR), (4) two stripes and two SRs, and (5) two stripes and 1 open book-like resonator (designed by putting two SRs obliquely) on Gallium Arsenide (GaAs) substrate (dielectric constant 12.96) (Fig. 1). GaAs substrate is selected due to its high band gap, high breakdown voltage, and better heat & moisture resistivity. A thin gold layer on the lower side of the substrate as a ground plane is used to suppress the transmission of the THz signal. We select gold for resonators due to its high conductivity, which causes inductance and capacitance to produce resonance at the desired frequency.

The highest absorption coefficient is obtained for an open book-type metastructure, where oblique sheets offer the ability to manipulate the bandwidth of MPA leading to different resonance conditions and different optical paths for resonances. The experimental method for fabrication of Multiple Oblique Flat Sheets (MOFS) based MPAs has been reported by Lu et al.^46^. They used e-beam lithography procedure where they fixed their sample on a sample holder with a tilted angle of $86^{\circ }$ with respect to the horizon and conducted oblique angle deposition. A Similar method can be used for the fabrication of book-type metastructure. Sensor parameters for an open book-type metastructure are optimized as: periodicity $(P)= 70\mu m$, substrate thickness $(t_s) = 15\mu m$, width of metal stripes $(w_s)= 4.5\mu m$, length of metal stripes $(L_s)= 50\mu m$, gap between the metal stripes $(G)=40\mu m$, the thickness of ground metallic plane $(t_m)= 0.08 \mu m$, length(*L*) and width(*w*) of the open book resonator $=20\mu m$ and $15\mu m$, $g=5\mu m$ respectively. These tilted resonators (periodic open books) can outperform the previously reported MPAs without any additional burden.

A plane electromagnetic wave (THz) polarized along the x-axis is used to illuminate the proposed device. We applied unit cell boundary conditions in x- and y- directions, while the THz beam considered as floquet port mode (with 2 essential modes) incident normally along the z-direction. The simulation is performed with $\lambda /100$ cells, where $\lambda$ is wavelength of incident radiation. Total number of tetrahedrons as 812 145 along with the adaptive meshing. The schematic of an MPA-based biosensor with incident, reflected, and absorbed THz beam is shown in Fig. 1f. Simulations are carried out using commercial software CST Microwave Studio Suite in frequency domain. A finite element method-based algorithm is employed to obtain precise simulation results. To assure minimum reflection of THz beam from metamaterial surface i.e. $R(\omega )=S_{11}^2 \approx 0$, where $S_{11}$ is scattering parameter that measures reflectivity. A perfect impedance matching with air intrinsic impedance ($Z_o$) (where real and imaginary parts of $Z(\Omega )$ are close to 1 and 0 respectively) is obtained. To suppress the transmission of THz wave through MPA to zero, a metallic ground plane on the bottom side of substrate $(T (\omega )=S_{21}^2=0$, where $S_{21}$ is scattering parameter that measures transmissivity) is used.

**Figure 2:**
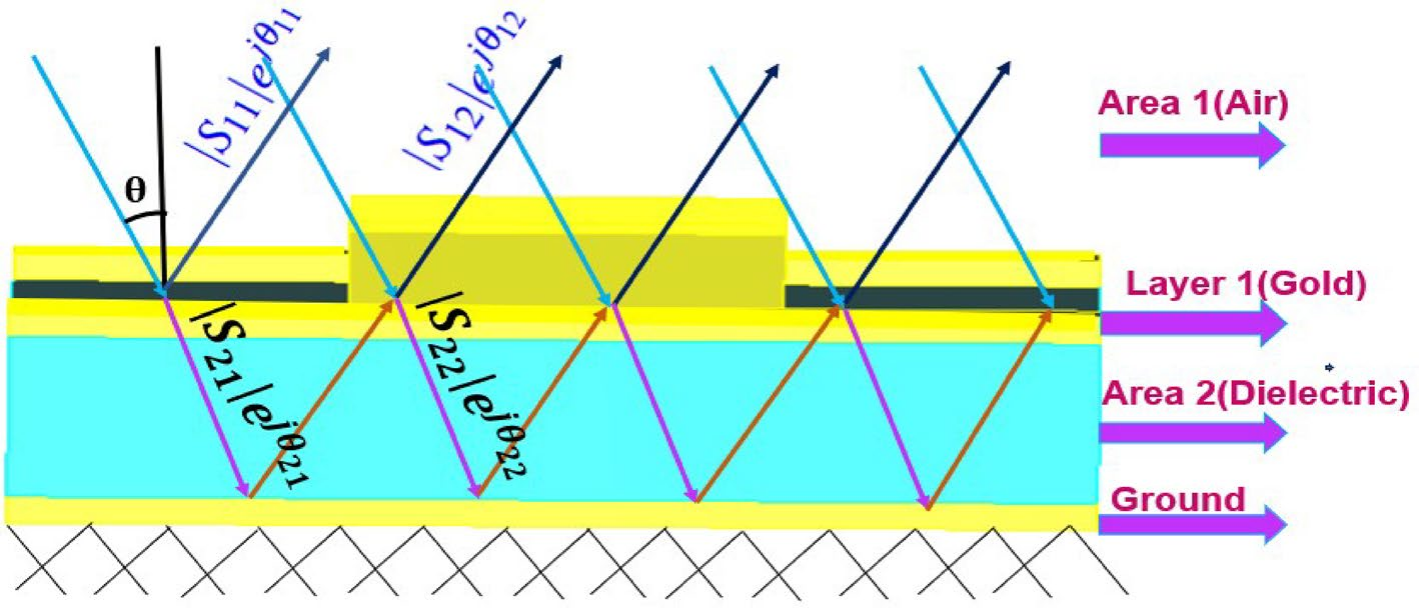
Schematic of interference theory model with incident, reflected, and transmitted wave.

The absorption of MPA can be explained using interference theory^47–49^. In Fig. 2, we considered the thickness of metallic layer as zero (denoted as layer 1). Air and dielectric substrate are denoted by area 1 and 2, respectively. The reflection coefficient of layer 1 i.e. area 1 to 1 is $S_{11}=| S_{11}|e^{j \theta _{11}}$, the transmission coefficient of layer 1 from area 1 to 2 is $S_{21}=| S_{21}|e^{j \theta _{21}}$, the transmission coefficient of layer 1 from area 2 to 1 is $S_{12}=| S_{12}|e^{j \theta _{12}}$, the reflection coefficient of layer 1 from area 2 to 2 is $S_{22}=| S_{22}|e^{j \theta _{22}}$. THz wave is incident in the positive z-direction, and metallic ground with boundary condition electric $(E_t=0)$, which work as a reflector is applied in negative z-direction. Total reflection coefficient $(S_{11total})$ for layer 1 is calculated as

1 $$\begin{aligned} S_{11total}&= S_{11} + S_{12} e^{ - j\beta } e^{ - j\pi } e^{ - j\beta } S_{21} + S_{12} e^{ - j\beta } e^{ - j\pi } e^{ - j\beta } S_{21} (S_{22} e^{ - j\beta } e^{ - j\pi } e^{ - j\beta } )^{1} S_{21} \nonumber \\&\quad + S_{12} e^{ - j\beta } e^{ - j\pi } e^{ - j\beta } S_{21} (S_{22} e^{ - j\beta } e^{ - j\pi } e^{ - j\beta } )^{2} S_{21} + \cdots \end{aligned}$$

2 $$\begin{aligned} S_{11total}&= S_{11} + S_{12} e^{ - j(2\beta + \pi )} S_{21} \sum \limits _{n = 0}^{\alpha } {\left( {S_{22} e^{ - j(2\beta + \pi )} } \right) ^{n}}\nonumber \\ S_{11total}&= \left| {S_{11} } \right| e^{{j\theta _{11} }} + \left| {S_{12} } \right| \left| {S_{21} } \right| e^{{ - j( - \theta _{12} - \theta _{21} + 2\beta + \pi )}} \big /1 - \left| {S_{22} } \right| e^{{ - j( - \theta _{22} + 2\beta + \pi )}} \end{aligned}$$

where $$|{S_{12}}| \approx |{S_{21}}|$$. Thus

3 $$\begin{aligned} S_{11total} = \left| {S_{11} } \right| e^{{j\theta _{11} }} + \left| {S_{12} } \right| ^{2} e^{{j(2\theta _{12} - 2\beta - \pi )}} \big /1 - \left| {S_{22} } \right| e^{{j(\theta _{22} - 2\beta - \pi )}} \end{aligned}$$

Here, $\beta =kd$ is propagation phase constant, d is the transmitted wave propagation distance from layer 1 to the ground and k is the wavenumber. We use these equations to calculate absorption of proposed MPA. Due to metallic ground, transmission coefficient becomes zero and absorption depends only on reflection coefficient $(S_{11total})$. We calculate the absorption coefficients of MPA as

4 $$\begin{aligned} A(\omega )=1-R(\omega )= 1 - | {S_{11total}} |^2=1 -\left| \frac{ Z_{in} -Z_0}{ Z_{in} +Z_0} \right| ^2 \end{aligned}$$

$Z_{in}$ (impedance of MPA) depends on its effective permittivity and permeability. Effective permittivity and permeability are the functions of electric and magnetic responses of MPA at resonance frequencies. In terms of S- parameters, $Z_{in}$ is calculated as

5 $$\begin{aligned} Z_{in}=\sqrt{\frac{\mu }{\epsilon }} =\sqrt{\frac{(1+S_{11})^2-S_{21}^2}{(1-{S_{11})^2- S_{21}^2}}} \end{aligned}$$

The absorption coefficients in terms of S-parameters are calculated as

6 $$\begin{aligned} A(\omega )=1-\left| {\frac{\sqrt{\frac{(1+S_{11})^2-S_{21}^2}{(1-{S_{11}})^2- S_{21}^2}}- Z_0}{\sqrt{\frac{(1+S_{11})^2 -S_{21}^2}{(1-{S_{11}})^2- S_{21}^2}}+Z_0}}\right| ^2 =1 -\left| \frac{\sqrt{(1+S_{11})^2}-Z_0\sqrt{(1-{S_{11}})^2}}{\sqrt{(1+S_{11})^2} + Z_0\sqrt{(1-{S_{11}})^2}}\right| ^2 \end{aligned}$$

**Figure 3:**
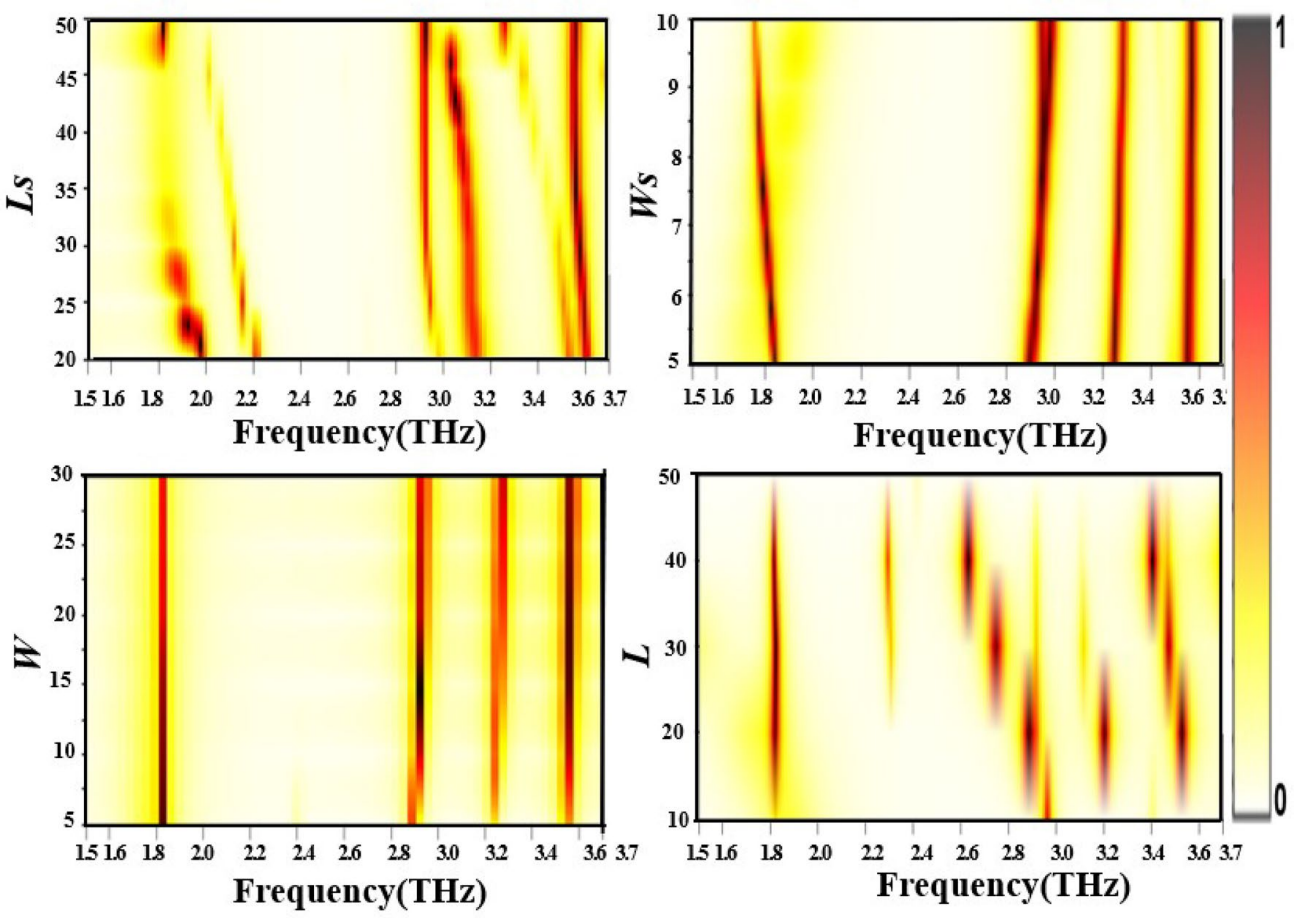
Absorption characteristics for different (**a**) length of metal stripe, (**b**) width of metal stripe, (**c**) width of open book resonator, and (**d**) length of open book resonator.

To better understand the absorption behavior, we thoroughly investigate the effect of structural parameters at resonance frequencies. 2D colormaps in Fig. 3 show the effect of variations in length of the stripe $(L_s)$, the width of the stripes $(w_s)$, width and position of the open book resonator on absorption coefficients. Value of $L_s$ is varied from $20\mu m$ to $50\mu m$ as shown in Fig. 3a. Due to the increase in inductance and capacitance of metallic stripes with an increase in $L_s$, a redshift in the resonances is observed with increase in $L_s$. The maximum absorption is obtained at $L_s=50\mu m$.

The effect of the width of metal stripe $(w_s)$ (from $4\mu m$ to $10\mu m$) on absorption coefficient is shown in Fig. 3b. The absorption coefficients for all four resonances become unity at $w_s=4.5\mu m$, which decreases with increasing $w_s$. Enhancement in the size of metallic resonators leads to high reflections. Absorption performance of MPA with width of open book type SRs(for $w=5-30 \mu m$) is shown in Fig. 3c. We obtain the highest absorption for $w=15\mu m$ without any shift in the resonance frequencies. Figure 3d shows the effect of the length of SRs (for $L=10$ to $50 \mu m$) on resonance frequencies and absorption coefficients. It is found that the absorption coefficient decreases with increase in the length of the open book due to high reflections. MPA gives the highest absorption for $L=20\mu m$ and zero absorption for $L=50\mu m$.

### Advance design system (ADS) based equivalent circuit model

**Figure 4:**
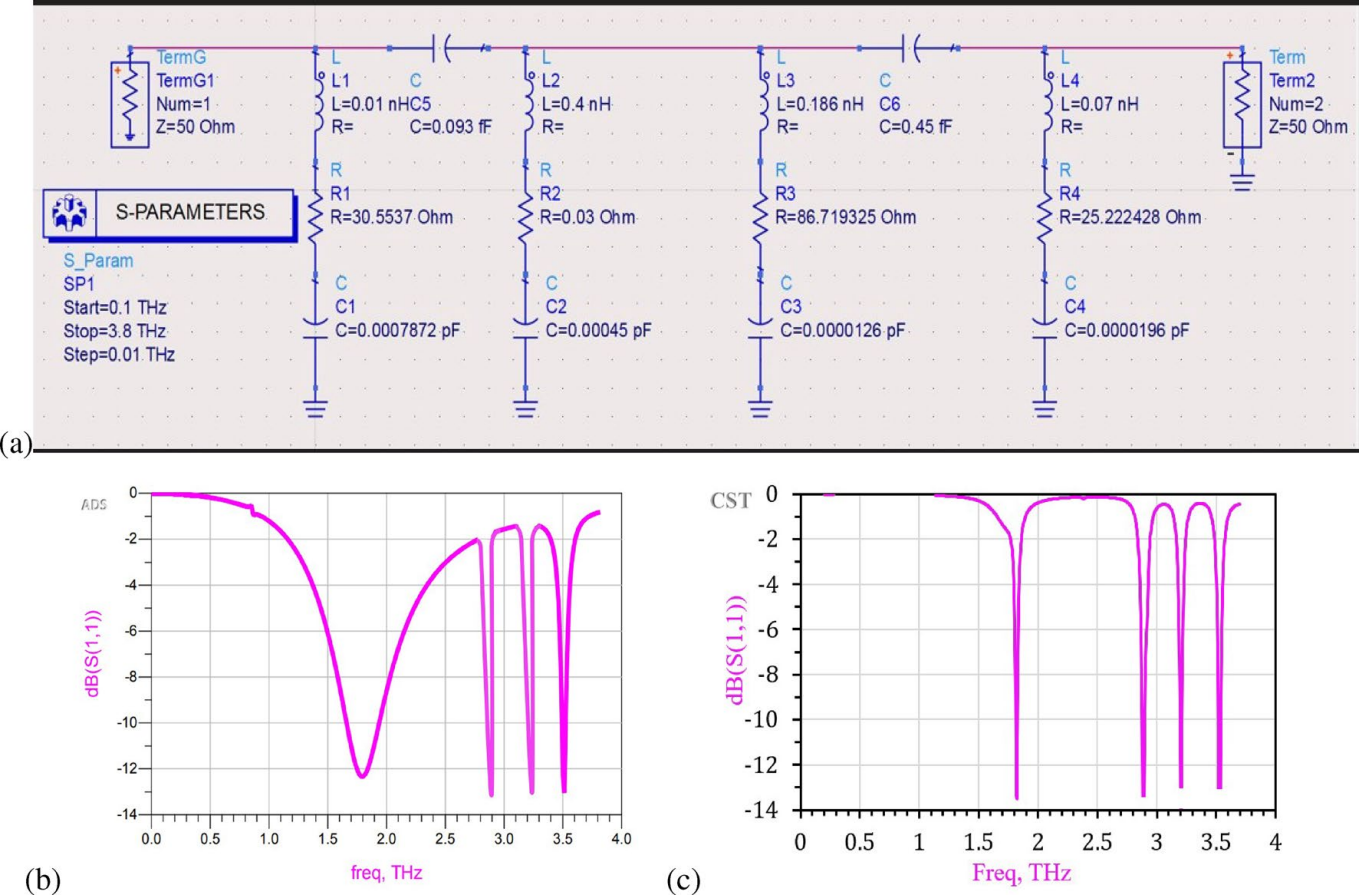
(**a**) Equivalent circuit design of MPA, (**b**) reflection coefficient spectra calculated by ADS, (**c**) reflection coefficient spectra simulated by CST.

We also design an equivalent lumped circuit using ASD -2019 simulation software, where unit cell of MPA has four resonators i.e. two metal stripes and two SRs as shown in Fig. 1e. Each resonator behaves as an RLC circuit (Fig. 4).

The inductances of each metallic resonator $$(L_1, L_2, L_3,$$ & $$L_4)$$ are calculated using Grover’s formula^50^

7 $$\begin{aligned} L= \mu _0\mu _r\left( ln \frac{2l}{t_m+w} -0.2235ln \frac{t_m+w}{l} +\frac{1}{2}\right) \end{aligned}$$

where *l* is the length of resonators, *w* is width of resonators, $t_m$ is thickness of resonators. The associated coupling capacitances $C_1, C_2, C_3, C_4$ of MPA is calculated using^51^

8 $$\begin{aligned} C_s= \epsilon _o\epsilon _r \frac{A_s}{d_s} \end{aligned}$$

where $A_s, d_s, \epsilon _o$ and $\epsilon _r$ represent the area of the stripe, capacitive gap between the resonators, permittivity of free space, and relative permittivity of the medium, respectively. We get similar reflection spectra of MPA as obtained in CST microwave studio suite-based simulations. The reflection characteristics $(S_{11})$ obtained by ADS perfectly match with simulation results (Fig. 4b,c), which confirms the accuracy of the results.

## Results and discussion

**Figure 5:**
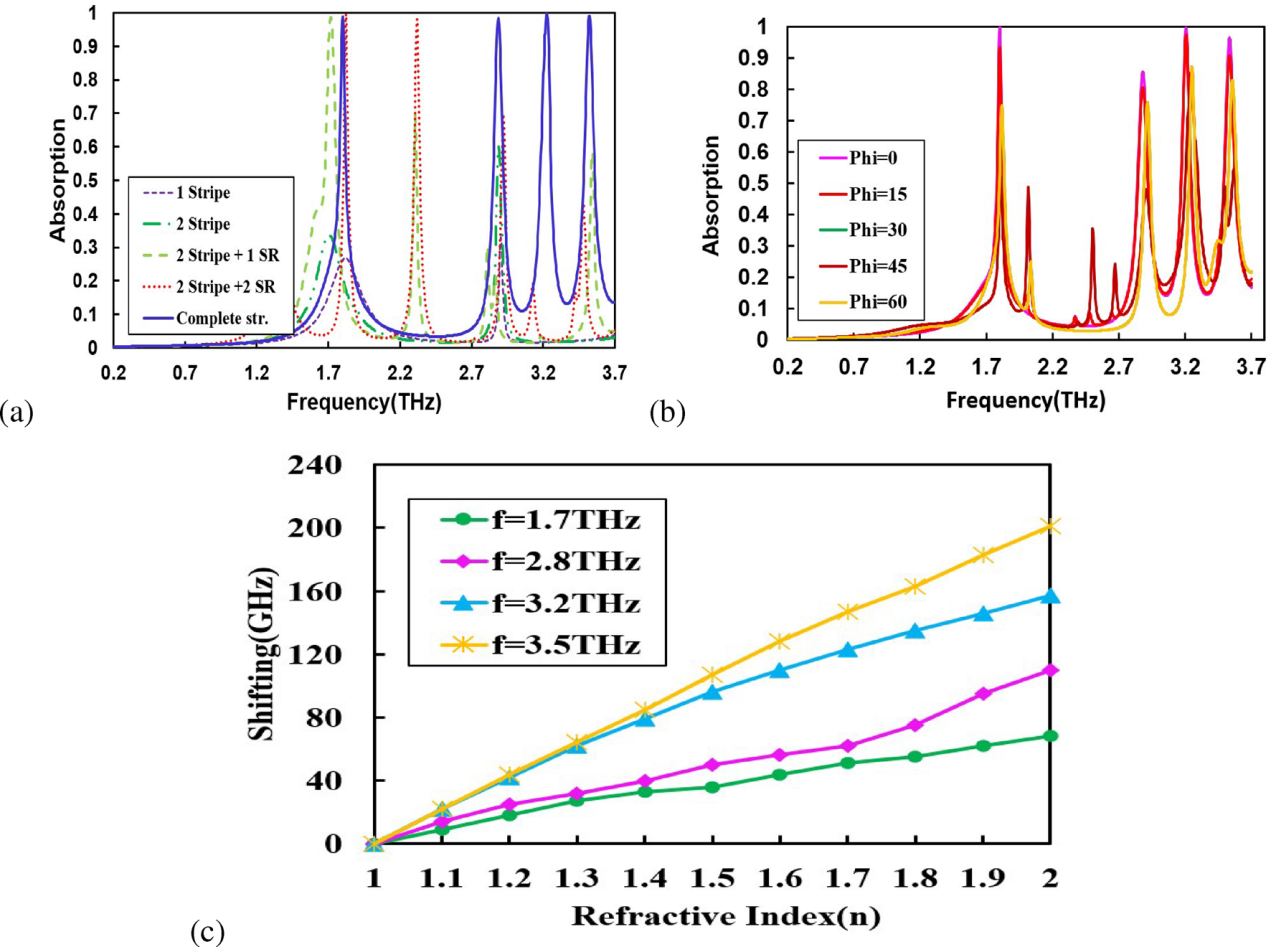
(**a**) The simulated absorbance spectra for five different types of resonators; (**b**) Absorption spectra for different incident angles of THz beam, and (**c**) Shifting in resonances with analytes of different refractive indices (1–2).

Prospects of MPA increase with the widening of working frequency band and also with the increase in the number of absorption peaks. We properly optimize the lattice structure (by optimizing the shape and size of the resonators) of the MPA to enhance the working frequency band and also the number of absorption peaks. We carry out simulations for absorption spectra for five different structures for : (1) one stripe resonator - we get two resonance frequencies with a maximum of $32\%$ absorption, (2) two stripes resonators- two resonances are obtained but absorption increases up to $50\%$, (3) two metal stripes and one square resonator (SR)- we get four absorption peaks, first with $99.12\%$ absorption, second with $70\%$ absorption, third with $58\%$ absorption, and fourth with $55\%$ absorption, (4) two metal stripes and two SRs- we get four absorption peaks, first & second with $99.12\%$ and $99.95\%$ absorption, third with $67\%$ absorption, and fourth with $42\%$ absorption, and (5) two metal stripes and one open book like resonator between the stripes –four resonance absorption peaks at frequencies $f_1= 1.7THz, f_2= 2.8THz, f_3=3.2THz,$ and $f_4=3.5THz$ with $99.12\%, 99.95\%, 98.5\%,$ and $99\%$ absorption are achieved as shown in Fig. 5a. The high absorption coefficients in case of book-like structure occur due to the strong inductive–capacitive coupling among the resonators. The resonators of different sizes are responsible for producing absorption peaks at specific frequencies and their superposition effect yields perfect absorption in multiple frequency bands^52–54^. Thus, the dimension of the unit cell structure and the shape of resonators have a vital role in accomplishing absorption near unity.

We also figure out the effect of incidence angle on the absorption peaks. Absorption spectra with different angles of incidence of THz wave are shown in Fig. 5b. As the incidence angle increases, the first resonance absorption peak (1.79*THz*) is almost stable while higher-order absorption peaks display slight deviations in resonance frequencies, which shows that lower frequency side resonance is stable with incident angle. The higher frequency side absorption peaks are slightly more sensitive to the incidence angle of THz than the first absorption peak. Figure 5c shows shifting in resonance frequencies after application of analytes of different refractive indices. The red shift in all four resonant frequencies is obtained with a change in the effective refractive index of the meta-surface environment. The redshift increases with an increase in refractive index of the testing sample materials. It is also observed from Fig. 5c that the red shift is larger for higher frequency resonances than the lower frequency resonances. Thus, it can be used to identify the type of analyte materials. The resonance shift is obtained as $S=f_a - f_o$, where $f_a$ is the resonance frequency with analyte refractive index and $f_o$ is resonance frequency without analyte(air). We get maximum frequency shift of 68*GHz*, 110*GHz*,  and 157*GHz*, 201*GHz* for $f_1= 1.7THz, f_2= 2.8THz, f_3=3.2THz, f_4=3.5THz$, respectively for refractive index 2. The highest shifting for $f=3.5THz$ is due to the fact that resonance shifting is greater for higher resonant modes^12^.

**Figure 6:**
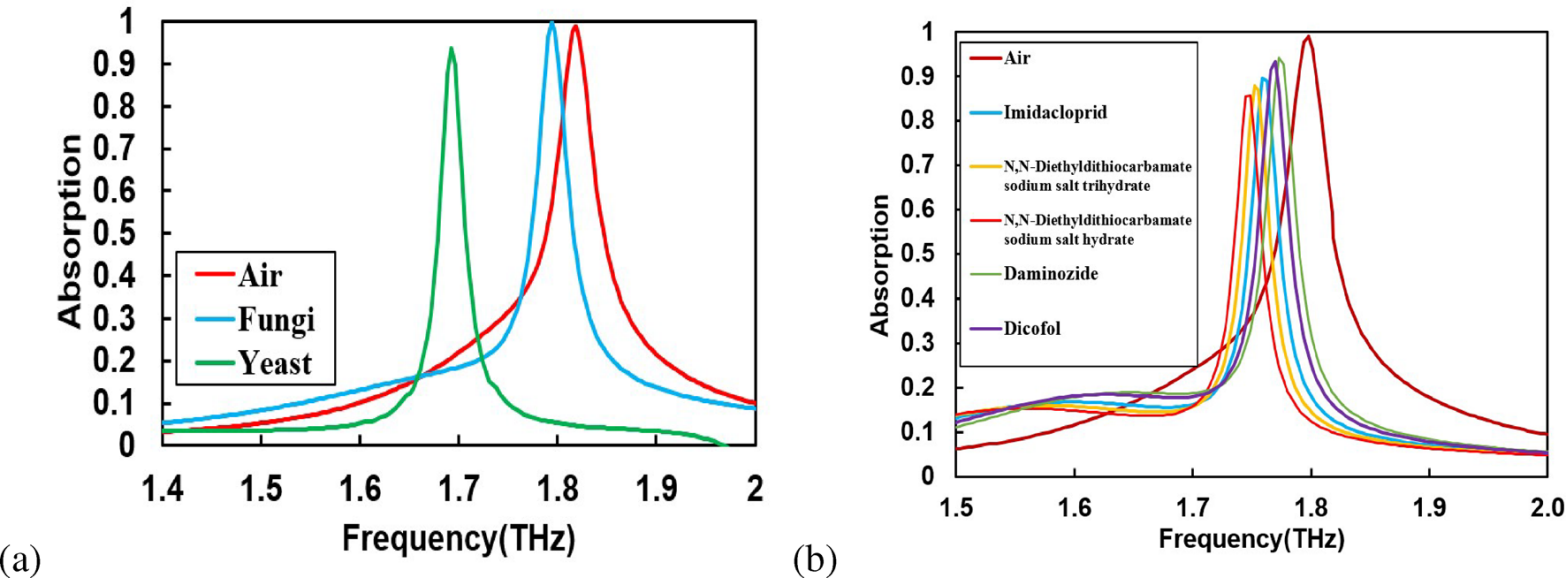
(**a**) Absorbance spectra for Penicillium chrysogenum(blue), yeast(red), and air (green) with the effective dielectric constant of 1.94, 8.2, and 1, respectively, and (**b**) Absorbance spectra for five types of wheat pesticides.

We investigate the performance of the sensor for different microorganisms such as yeast, pesticides, and fungi. Experimental data reported by Park et al.^16^ and theoretical data (obtained using the effective medium theory based on the Maxwell-Garnett mode) for dielectric constants of microorganisms are used for our investigations. Experimentally, Penicillium chrysogenum (fungi) and yeast were grown by streaking on medium method at $37^{\circ } C$ and deposited on the surface of the metamaterial. A conventional photo-lithography method was used to prepare the metamaterial pattern and the THz time-domain spectroscopic technique was used to characterize the samples^16^. The measured values of the dielectric constants were reported between $1.37-1.94$ for fungi and $5.38-8.54$ for yeast. Experimental findings were examined using the finite difference time-domain solver. The absorption patterns for Penicillium chrysogenum(fungi) and yeast are shown in Fig. 6a. The respective frequency shifts of 70*GHz* and 103*GHz* are observed for Penicillium chrysogenum and yeast. Due to large dielectric constant of yeast, redistribution of the resonance of electric and magnetic fields leading to a strong modulation of the absorption peak provides a larger resonance shift. The traits of the sensed microorganism can be obtained by calculating the frequency shift of the absorption peaks.

We also study the sensing performance for several pesticides like Imidacloprid, N, N-Diethyldithiocarbamate sodium salt trihydrate, Daminozide, N, N-Diethyldithiocarbamate sodium salt hydrate, and Dicofol. For this purpose, we use the experimental values of refractive indices reported by Maeng et al.^6^. The measured refractive index(RI) of Imidacloprid, N, N-Diethyldithiocarbamate sodium salt trihydrate, Daminozide, N, N-Diethyldithiocarbamate sodium salt hydrate, and Dicofol are shown in Table 1. The absorption vs. frequency spectra of all pesticides are shown in Fig. 6b. A redshift of resonance frequencies by 95*GHz* is observed in the presence of pesticides. A comparison of sensing performances with earlier reports is given in Table 2. The comparison shows that the sensitivity of our sensor is multi-fold higher for pesticides and microorganisms than previously reported sensors^15, 16, 55–57^.

**Table 1:** Name of pesticides used for sensing, along with their molecular formula, refractive index, and absorption peak in THz region.

Name of pesticides	Formula	RI	Absorption (THz)
Imidacloprid	$C_9H_{10}CIN_5O_2$	1.87	1.36,1.76
N,N-Diethyldithiocarbamate sodium salt trihydrate	$C_5H_{10}NNaS_2*3H_2O$	1.99	1.11, 1.41, 1.74, 2.40
N,N-Diethyldithiocarbamate sodium salt hydrate	$C_3H_6NNaS_2*H_2O$	2.08	0.99, 1.50
Daminozide	$C_6H_{12}N_2O_3$	1.68	1.32, 2.40
Dicofol	$C_{14}H_9C_{15}O$	1.70	2.34

**Table 2:** A comparison of sensing performance of proposed THZ MPA sensor for pesticides with previously reported sensors.

Reference	Frequency (THz)	Resonaces (THz)	Sensing target	Shifting (GHz)
Tantiwanichapan et al.^44^	1.8–2.4	1.92	Pesticides	20
Xu et al.^45^	0.75–0.95	0.86	Pesticides	15
Cui et al.^46^	0.4–2.4	1.03, 1.45, 1.77	Pesticides	40
This work	0.2–3.7	1.7, 2.8, 3.2, 3.5	Microorganisms	103
This work	0.2–3.7	1.7, 2.8, 3.2, 3.5	Pesticides	95

**Figure 7:**
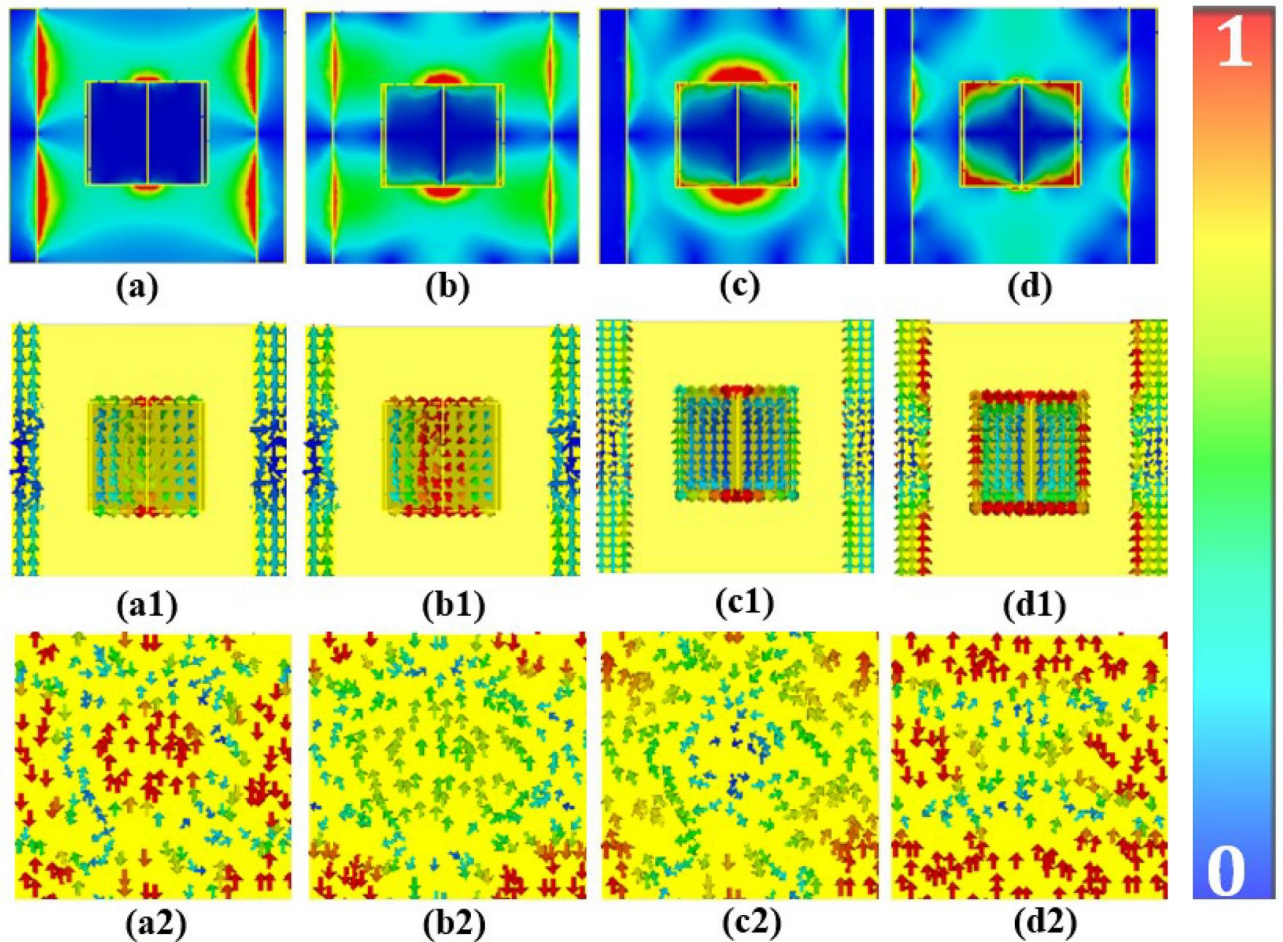
(**a**–**d**) Electric field, (**a1**–**d1**) Surface current distributions on top layer, and (**a2**–**d2**) Surface current distributions on bottom layer of the designed MPA at resonance frequencies: $(a1, a2) f_1= 1.7 THz, (b1,b2) f_2 = 2.8THz, (c1,c2) f_3 =3.2 THz, (d1,d2) and f_4 = 3.5THz$.

To gain deep insight into the physical mechanism behind the absorption process of MPA, we study the electric field and surface current distribution of a unit cell at resonance conditions. Figure 7a–d show the simulation results of the electric field at resonances 1.7*THz*, 2.8*THz*, 3.2*THz*,  and 3.5*THz*, respectively. Figure 7a1–d1 & a2–d2 show the surface current distributions of the top and the bottom layers, respectively. Antiparallel currents form a current loop leading to the excitation of the magnetic dipole resonance^58^. The surface currents on both metallic layers are flowing in opposite directions at resonances (Fig. 7), which generates the magnetic flux coupling with the incident H-field. The antiparallel surface currents on the top and bottom layers of MPA validate that the absorption peaks are caused by magnetic resonance responses. Therefore, the stronger absorption of the MPA is instigated by the excitation of multiple high-order magnetic resonances.

## Conclusion

We develop a novel THz MPA-based sensor for sensing biological analytes. The highest absorption coefficient is obtained for an open book-type metastructure. Four resonance absorption peaks at frequencies $f_1= 1.7THz, f_2= 2.8THz, f_3=3.2THz,$ and $f_4=3.5THz$ having absorption coefficients $99.12\%, 99.95\%, 98.5\%,$ and $99\%$, respectively are obtained.We also studied the absorption spectra of the MPA with different oblique incidences of THz wave and found that lower resonance absorption peak (1.79*THz*) is almost stable while higher absorption peaks show slight deviations. The effect of structural parameters(length and width of metal stripes and book resonator) on the resonance frequencies is analyzed to get the optimum performance of the MPA. To gain deep insight into the mechanism of MPA, an equivalent circuit model is developed using advance design system (ADS) software. Multiple interference theory is used to explain the absorption mechanism. Surface current distribution depicts that strong absorption of the MPA is instigated by the excitation of multiple high-order resonances. Due to the change in the effective dielectric constant of the MPA environment with the deposition of microorganisms, significant redshifts in the resonance peaks (redshift of 103*GHz* for yeast, redshift of 70*GHz* for fungi) are observed. These shifts in resonance frequencies are due to the redistribution of the resonance electric and magnetic fields, which lead to a strong modulation of the absorption. Thus, by functionalizing the substrate with antibodies, a selective detection of microorganisms is also plausible. We believe that this MPA-based THz biosensor can open up new opportunities for obtaining dielectric information on microorganisms, which has great potential in label-free biosensing for future biomedical applications.

## Methods

We have used commercial software CST Microwave Studio Suite to design and simulate our MM unit cell. In x – and y – directions, unit cell boundary conditions are applied and in z-direction, open add space is employed to obtain the absorption behavior of the unit cell with adaptive tetrahedral messing. THz radiation is incident normally on the structure with an electric field along the x-direction. A field monitor is applied at the resonant frequency to obtain electric field and surface current distribution. Additionally, advanced design system(ADS -2019) software is used to design an equivalent circuit of MPA using to analyze its functioning. In our simulation, we chose GaAs as a substrate with a dielectric constant of 12.94 and loss tangent of 0.006, and gold as a metal for resonator^18^.

## Data Availability

The datasets used and/or analyzed during the current study are available from the corresponding author upon reasonable request.
